# Transdermal Drug Delivery Systems: A Focused Review of the Physical Methods of Permeation Enhancement

**DOI:** 10.34172/apb.2024.018

**Published:** 2023-10-14

**Authors:** Rifath Sheikh Vaseem, Alison D’cruz, Srishti Shetty, Hafsa -, Aditya Vardhan, Shreya Shenoy R, Shirleen Miriam Marques, Lalit Kumar, Ruchi Verma

**Affiliations:** ^1^Department of Pharmaceutics, Manipal College of Pharmaceutical Sciences, Manipal Academy of Higher Education, Manipal 576 104, Udupi, Karnataka, India.; ^2^Department of Pharmaceutics, National Institute of Pharmaceutical Education and Research, Hajipur 844 102, Vaishali, Bihar, India.; ^3^Department of Pharmaceutical Chemistry, Manipal College of Pharmaceutical Sciences, Manipal Academy of Higher Education, Manipal 576 104, Udupi, Karnataka, India.

**Keywords:** Transdermal, Advanced techniques, Facilitated transdermal drug delivery, Transcellular mechanism

## Abstract

The skin is the body’s largest organ and serves as a site of administration for various medications. Transdermal drug delivery systems have several advantages over traditional delivery systems. It has both local and systemic therapeutic properties. Controlled plasma drug levels, reduced dosing frequency, and avoidance of hepatic first-pass metabolism are just a few of these systems’ advantages. To achieve maximum efficacy, it is critical to understand the kinetics, physiochemical properties of the drug moiety, and drug transport route. This manuscript focused on the principles of various physical means to facilitate transdermal drug delivery. Some examples are iontophoresis, electrophoresis, photomechanical waves, ultrasound, needleless injections, and microneedles. Mechanical, chemical, magnetic, and electrical energy are all used in physical methods. A major advantage of physical methods is their capability to abbreviate pain, which can be used for effective disease management. Further investigation should be carried out at the clinical level to understand these methods for effective drug delivery.

## Introduction

 Although oral drug delivery works well for medications with strong epithelial permeability and aqueous solubility, it can be challenging to administer weakly water-soluble medications.^1^ The limitations of the oral route, such as hepatic first-pass metabolism leading to low oral bioavailability, high frequency of dosages, large doses, and others, necessitated the introduction of alternate routes of administration to increase bioavailability and eliminate the physiological barriers brought on by the unique physiological milieu, such as pH, bile salts, enzymes, intestinal motility, and hydrodynamics.^2^ Given the motives to exploit new delivery routes, numerous studies describe the utilization potential for adequate bioavailability and marketed products for the various routes of administration.^3^ They can deliver novel, genetically modified pharmaceuticals to the site of action without altering their intended effect.^4^

 The skin, the first line of protection for the body, is the largest organ for drug delivery, but its primary function restricts its efficacy for this purpose.^5^ The stratum corneum (SC), the skin’s outermost defense against foreign objects, prevents the transdermal passage of drugs.^6^ Drug delivery across the skin influences tissues lying in the neighborhood of the administration site and has an effect when distributed in the systemic circulation.^7^ Transdermal drug administration offers a plethora of benefits over oral and hypodermic injections.^8^ The delivery of numerous therapeutic substances has been dramatically impacted by transdermal delivery, particularly in treating cancer, diabetes, cardiovascular and central nervous system disorders.^9-12^ In 1979, the Food and Drug Administration (FDA) authorized the first transdermal delivery device, a patch to cure motion sickness. Later, different strategies were developed, such as structure-based, electrical-based, and velocity-based strategies. These innovations were made to surmount the difficulties and challenges of administering the drug using this technique, which has enhanced patient compliance.

###  Advantages of the transdermal drug delivery system

- Due to the continual advancements in innovation plus the capability to deliver the medication to the site of action without disrupting the skin, transdermal delivery is becoming one of the foremost acknowledged methods of drug delivery.^4^
- Escaping drug exposure to the gastrointestinal tract (GIT), hepatic first-pass metabolism, enzymatic breakdown, and gastrointestinal discomfort.^13^
- Improves drug absorption and maintains a constant drug concentration in blood for a predefined duration.
- Easy to scale up. Several drugs are commercially available as transdermal patches.^13^
- Improves patient compliance.
- Reduced inter- and intrapatient variability.^4^
- Decreases dose to be administered.
- Minimizes gastrointestinal (GI) side effects.
- It is easy to discontinue the treatment in case of toxicity or side effects.
- Drug administration is possible in the case of unconscious patients.
- Having a relatively large area of application compared to the buccal or nasal cavity.

###  Disadvantages

- Transdermal drug delivery systems are incapable of conveying ionic medications/drugs.^14^
- Drugs with a size greater than 500 Daltons are not appropriate for transdermal delivery.^15^
- It is not capable of reaching elevated drug levels in blood/plasma.
- Possibility of skin irritation, erythema, and itching.
- It cannot transmit drugs in a pulsatile fashion.
- Long-term adherence causes discomfort to the patient.^15^
- To traverse the SC and the underlying aqueous layer, adequate solubility in the hydrophilic and hydrophobic phases and a log P between 1 and 3 are necessary.
- Drugs with low or high partition coefficients cannot enter the bloodstream.^15^
- Lesser dose candidates are preferred for this type of drug delivery system.

## Skin and permeation

 The skin is the body’s largest organ, with a surface area of approximately 2 square meters, and obtains approximately one-third of the overall blood supply.

 Its functions are as follows:

- It protects from physical, chemical, and biological attacks and environmental conditions.
- It works as a permeability barrier, enabling biological and chemical substances to be absorbed transdermally.^14,16^

**Figure 1:**
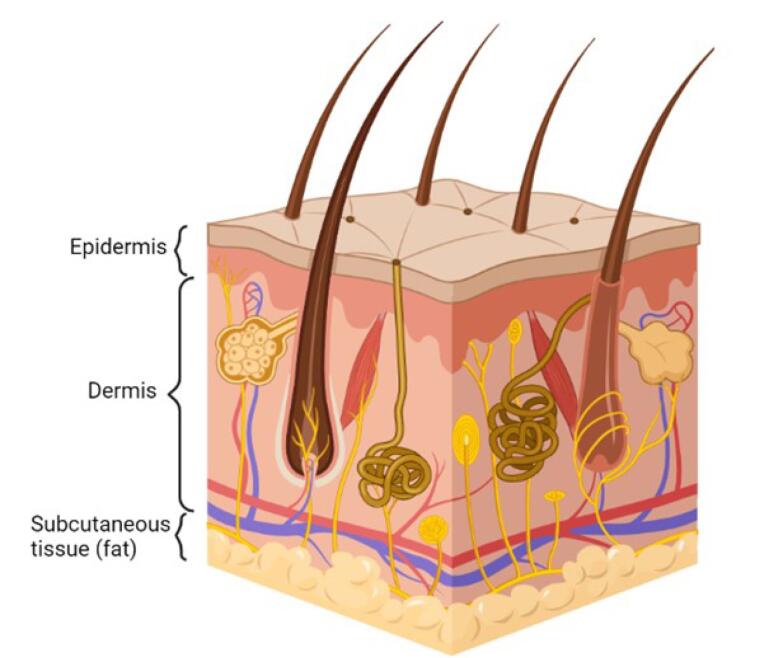
Physiology of skin

There are three main regions of the skin, i.e., epidermis, dermis, and hypodermis. As seen in Figure 1, the epidermis is the outermost layer of the skin, which covers the whole body’s surface. It is a self-regenerating and stratified squamous epithelium that comprises a nonviable epidermis called the SC and a viable epidermis separated into four layers, i.e., the stratum lucidum, stratum granulosum, stratum spinosum, and stratum germinativum.^13,14^ The SC, which is composed of 15-20 layers of keratinocytes bound to a lipophilic matrix, is the rate-limiting barrier in the transdermal permeability process.^13^ The dermis is the skin’s middle layer, approximately 2-3 mm thick. It comprises 70% collagenous tissues and elastic fibres, giving the skin strength and elasticity. It also comprises different blood vessels, nerves, and lymph vessels. It possesses a negligible hurdle to the transdermal entry of polar drugs.^14,16^ The subcutaneous layer is the innermost layer of the skin and consists of fat cells. It helps to maintain body temperature and provides nutritional support and mechanical strength to withstand physical shock.^4^ A transdermal drug delivery system must penetrate all three layers of the skin for medication delivery to the systemic circulation.

###  Permeation process

**Figure 2:**
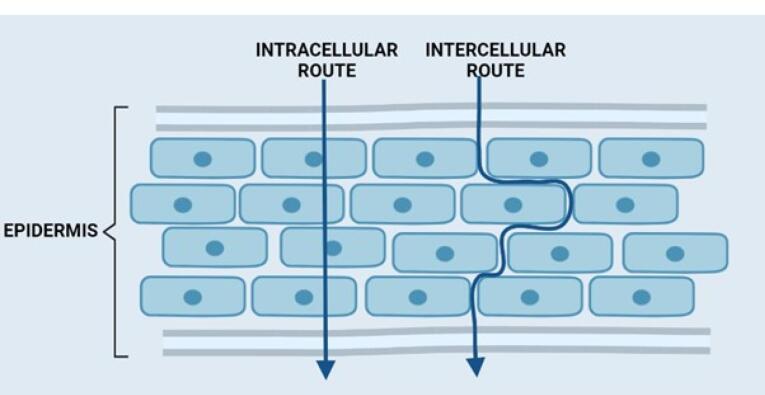
Routes of transdermal permeation

 The transdermal drug can be delivered through the skin into circulation by three different routes (Figure 2).

####  Trans-appendageal route

 This is also referred to as the SHUNT route. Permeation occurs across the hair follicles through the sweat glands and sebaceous glands. The presence of these appendages provides a clear path across the SC. The follicular number, volume, and opening width are all significant factors in the transport of drugs via appendages.

####  Transcellular route

 Although it is thought to be the quickest pathway, drug molecules face significant obstacles since they must go beyond both the hydrophilic and hydrophobic structures.^17^ A candidate to cross via this route must partition into and diffuse through the corneocytes.^17-20^ Small lipophilic molecules after partitioning in cell membranes can penetrate via this route.^21^ Hydrophilic molecule partitioning is limited in the cell membrane; however, such types of molecules can penetrate via this pathway if their size is small and/or receptor-mediated transporters are used.^21^

####  Intercellular route

 This route is also known as the paracellular route. Hydrophilic drugs can diffuse via this route; however, a smaller particle size is preferred for this pathway. The diffusion of drugs is limited owing to the occurrence of tight junctions.^14,16,21^

## Components of transdermal drug delivery systems – especially transdermal patches

**Figure 3:**
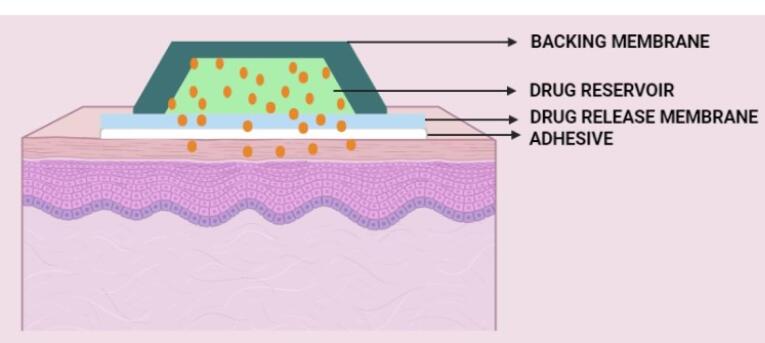
Components of the transdermal drug delivery system

 A transdermal patch usually consists of the following components (Figure 3):

- Liner
- Drug
- Adhesive (pressure-sensitive adhesive)
- Membrane (polymer matrix/drug reservoir)
- Backing laminates
- Other excipients include permeation enhancers, plasticizers, and solvents.

###  Liner

 It is a form of primary packaging that is removed during the application of a transdermal patch on the skin. The primary purpose of the liner is to safeguard the dosage form during storage.^20^ The liner must be chemically inert and should not release or interact with the transdermal patch. Usually, the liner is made up of a base that may be non-occlusive (e.g., paper fabric) or occlusive (e.g., polyethylene, polyvinyl chloride). In contrast, the release coat is composed of Teflon or silicon. Materials employed for release liners also consist of polyester foil and metalized laminates.^13^

###  Drug

 The transdermal system is a preferable choice for drugs that experience high first-pass metabolism and the highest degradation in the GI tract, which has a narrow therapeutic window, and drugs having a short half-life ( < 2 hours) that results in frequent dosing and finally causes non-compliance by the patient.

 The following properties should be considered for drugs that are administered through the transdermal route:

- Drug must have a molecular weight less than 500 Da.
- Drug must possess an affinity towards hydrophobic as well as hydrophilic phases.
- Drug should have a low melting point.
- Drugs must have a lower daily dose (preferred less than 20 mg), must be non-irritant and non-allergic and must possess a short half-life.
- The log k partition coefficient of the drug is preferred between 1 and 4.
- Drug solubility of more than 1 mg/mL is preferred between pH 5.0 and 9.0.

###  Adhesives

 The primary function of a pressure-sensitive adhesive is to maintain close proximity between the skin and delivery system. The physicochemical properties of adhesives generally depend on the types of excipients and the amount of these excipients, mainly the composition of the transdermal drug delivery system, adhesive layer thickness, thickness of the backing membrane, residual solvent, and active pharmaceutical ingredient concentration.^21^ An adhesive should possess the following characteristics^4^:

- It should be easily attachable without any damage to the tissue.
- It should be easily and completely removable without leaving any residue and should attach aggressively.
- It should not cause irritation or sensitization of the skin.
- It should be physically and chemically compatible with the drugs and excipients used in the drug delivery systems.
- The permeation of the drug should be unaffected.

###  Membrane (polymer matrix)

 The backbone of a transdermal delivery system is polymers, which control the medication’s release from the system. The drug can be dispersed in a synthetic polymer in solid or liquid form to create a polymer matrix. The polymers utilized in transdermal delivery should be biocompatible and chemically compatible with the drug, penetration enhancers, and adhesives. They must also distribute a drug consistently and effectively over the product’s stated shelf life and be safe.^13^

 Polymers utilized in transdermal delivery systems include the following:

- *Natural polymers: *e.g., Cellulose derivatives, zein, gelatin, shellac, waxes, proteins, gums, natural rubber, and starch.
- *Synthetic elastomers: *e.g., polysiloxane, polybutadiene, silicone rubber, hydrin rubber, neoprene, nitrile, acrylonitrile, styrene-butadiene rubber, and butyl rubber.
- *Synthetic polymers:* e.g., polyvinyl chloride, polyvinyl alcohol, polypropylene, polyethylene, polyamide, polyacrylate, polyurea, polymethylmethacrylate, etc.

The following criteria are considered for the selection of polymers for transdermal drug delivery systems:

- For the drug to diffuse and be released from the formulation, the molecular weight, glass transition temperature, and chemical functionality of the polymer must be considerable and must diffuse the drug properly and be released through it.
- The polymer must be nonreactive with the drug and biological system and must be stable in combination with the drug.
- It must be easy to manufacture into the desired product.
- It must be economical.
- The polymer and its degraded substances should not be toxic to the users.
- When high concentrations of active substances are introduced into the polymer, the mechanical characteristics of the polymer should not decrease drastically.^21^

###  Backing laminates

 When using the system and the transdermal patch, backing films are essential. The film’s job is to maintain the system’s stability, protect the active layer, and regulate skin permeation and tolerance based on breathability or occlusion.^22^

 While preparing backing laminates, the following characteristics should be considered:

- The material must be chemically resistant.
- It should be compatible with excipients, drugs, and enhancers to prevent leaching.
- It should have high flexibility or low modulus.
- It should be an impermeable substance that should protect the product during use on the skin.
- It should have good oxygen transfer and a high moisture transmission rate, e.g., vinyl, polyethylene, polyester, aluminum, and polyolefin films.^13^

###  Other components

*a) Permeation enhancer:* Penetration enhancers (also known as sorption promoters or accelerants) are a long-standing method for increasing transdermal delivery by increasing the permeability of the SC to achieve greater therapeutic concentrations of the active moiety. They play a crucial role in improving the flux (via thermodynamics - the concentration gradient, diffusion coefficient – molecular size and shape, and reducing the energy required to make a molecular hole in the membrane).^23,24^ Penetration enhancers interact with structural elements of the SC, causing the barrier properties to be altered and permeability to be increased. There are three ways of penetration of the drug through the skin, i.e., polar, non-polar, and polar/non-polar. In polar drugs, the enhancer alters the protein conformation or solvent swelling. However, the key to altering the non-polar route is to modify the rigid nature of the lipid layer and fluidization of the crystalline pathway. The fatty acid enhancer increases the fluid nature of the lipid structure of the SC. Several accelerants (binary vehicles) alter the multilaminate pathway for penetrants, thus helping both polar and non-polar pathways.

 Ideal characteristics for permeation enhancers are as follows:

- It should be biocompatible and must not cause toxicity.
- It should be compatible with the drug.
- It should be affordable, readily available and possess good solvent properties.
- The process should be reproducible, sustainable, and rapid.
- It should not promote leakage of body fluids or endogenous substances, i.e., it must have unidirectional flow, and it should promptly restore the skin’s natural barrier characteristics upon removal.^25^

*b) Plasticizer: *They impart flexibility and enhance the tensile strength of the film.^26^ They also influence the drug release, permeability, stability, elasticity (preventing film cracking), and wearing properties of the transdermal system. One of the most important advantages is to control the release rate of the drug, which could be obtained by selecting a particular type of plasticizer and optimizing its concentration in the preparation.

 Common plasticizers in transdermal patches are phthalate esters, fatty acid esters, and glycol derivatives.^27^

*c) Solvent:* The primary use of the solvent is to enhance the permeability, possibly by swallowing the polar pathway and/or fluidizing lipids. Examples: Alcohol- methanol and ethanol; alkyl methyl sulfoxides — dimethyl sulfoxide, and dimethyl formamide; pyrrolidones; laurocapram; miscellaneous – propylene glycol, glycerol, silicone fluids, isopropyl palmitate.

*d) Surfactant:* The main role of surfactants is to alter the pathway for polar transport, especially for hydrophilic molecules. These either enhance the compound partitioning between the formulation medium and the SC or higher compound diffusivity inside the SC could increase the transdermal absorption. Surfactants may change the saturation state of the medicine inside the formulation or interact with skin-related components.^28^ These compounds modify penetration using the polar head group and the hydrocarbon chain length. However, these compounds may be skin irritants. Hence, a clear balance between the penetration enhancement and irritation profile is suggested. Examples:

- *Anionic surfactants: *These can penetrate and produce more alteration after interacting strongly with the skin. e.g., Sodium lauryl sulfate, dodecylmethyl sulfoxide, dioctyl sulfosuccinate, etc.
- *Nonionic surfactants: *These are widely used due to their low irritation profile. e.g., Pluronic F68, Pluronic F127, Sodium tauroglycocholate, Sodium deoxycholate.
- *Binary system: *These surfactants function by opening heterogeneous multilaminate pathways together with continuous paths. e.g., 1,4-butane diol-linoleic acid and propylene glycol-oleic acid.^1^

## Recent advances in physical methods of permeation enhancement in transdermal drug delivery systems

 Technologies for physical enhancement have proliferated where chemical enhancement has reached its limits. We have made an effort to provide a general overview of the approaches used in the currently explored forms of physical transdermal distribution in the sections below. A summary of the advanced permeation techniques used in transdermal delivery is presented in Table 1.

**Table 1:** Advanced transdermal drug delivery techniques

**Advances in TDDS**	**Advantages**	**Disadvantages**	**Examples**	**References**
Iontophoresis	- Aids in the distribution of charged or neutral compounds through topical and transdermal routes.	- A more significant current intensity or the characteristics of the drug molecule can sometimes produce erythema or skin irritation.	LidoSite^TM^	^29^
	- Small peptides have traditionally been delivered by iontophoretic delivery.			
Electroporation	- Relatively safe and painless method that has been shown to administer LMW medicines successfully.	- A lot of cell disruption, including cell death and damage to heat-liable medicines is seen.	Transdermal medication of Piroxicam with two surfactants (Tween 80 and sodium lauryl sulfate).	^30,31^
	- The transdermal penetration rate and extent may be controlled by adjusting the electroporation settings.			
Photomechanical waves	- Delivers macromolecules.	- Poor understanding of the mechanism.	*In vivo* topical drug delivery of 5-aminolevulinic acid using a single photomechanical wave	^32,33,34^
	- Deeper penetration into the tissue.	- No human clinical data.		
	- Increase permeability of cell membranes.			
Ultrasound	- Increase delivery of high and low molecular weight drugs.	- Sophisticated technique.	Sonoprep®	^35,36^
	- Enhanced delivery through the transdermal route.	- Energy consuming.	Sonoderm^TM^	
	- Delivers drugs in a low-frequency range.	- Time consuming.		
	- Controlled dispersion.	- Irritation and burning sensation.		
	- Patient compliance.			
Microneedles	- Microbial penetration is reduced because, unlike a hypodermic needle, the microneedle punctures the epidermis.	- Dosage precision may be inferior to that of hypodermic needles.	MicroCor PTH	^37^
	- Rapid medication administration is achieved by combining the microneedles with an electrically controlled micropump.	- Repeated injections may cause vein collapse.	Corplex	
	- Compared to the drug delivery via the SC, the rate of drug delivery can be controlled more efficiently with this technique.	- Dermal tissue that is compressed can prevent hollow microneedles from functioning correctly.		
Suction abrasion	- Eliminates the discomfort due to dermal invasion.	- Prolonged period to reach a blister.	Cell patch	^38^
		- Can cause epidermal infections (less severe).		
Skin puncture	- Suitable for transferring drugs like insulin.	- Excessive bleeding, if not used properly.	Imprinter®	^39^
	- Faster action.	- Lightheadedness.		
		- Scarring (occurs when there have been multiple punctures in the same area).		
Needle-free injection technology	- No skin puncture or any type of damage to the skin.	- Not suitable for intravenous administration	Ped-O-Jet	^40^
	- Quicker drug delivery and improved reproducibility.	- The procedure is time-consuming and costly.		
	- Self-administration is possible due to needleless technology.	- Proper training and upkeep are needed.		
	- Improves vaccine response.			
	- Medication can also be administered in the form of dry powder.			
Radio-frequency microchannels	- Painless, safe and effective substitute to intramuscular or subcutaneous vaccines, allowing for expanded vaccinations.	- Maintenance of high-frequency alternating current (~ 100 kHz).	ViaDerm	^41^
	- Improve drug material penetration as well as dosing management.			
Magnetophoresis	- Induction of structural changes that could lead to enhancement in the permeability.	Alters the properties of the SC.	Lidocaine transdermal patch	^36,42^
	- Enhancement in permeant flux.			

###  Iontophoresis

**Figure 4:**
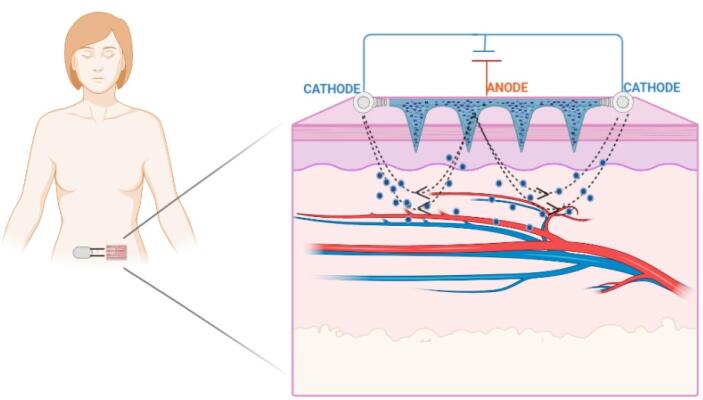
Diagrammatic representation of iontophoresis

 Iontophoresis is a holistic procedure that includes applying a low-voltage charge on the skin (Figure 4).

 This method improves the dispersion of neutral or charged particles on the skin surface and enhances transdermal delivery.^29^ A restricted, short-planned, and limited milliampere current (0.1–1.0 mA/cm^2^) is applied on the skin, and the medication is delivered to the whole and bound region beneath the skin layers.^30^ Iontophoresis enables the drug particles to penetrate across the skin by two primary methods: Penetration of charged particles due to electrical repulsion from the electrode and penetration of unionized particles by electro-osmosis. The trans-appendage route plays a fundamental role in drug transport via electro-osmosis and electromigration. During iontophoresis, particles lean towards the route with the least amount of electrical obstruction, such as hair follicles or sweat glands. For a drug particle to penetrate across the skin, it must preferably be hydrophobic and have a molecular weight of less than 500 Da.^29^ As per Faraday’s law, the iontophoretic flux of a drug particle (J_DRUG_) is the amount of the fluxes because of electromigration (J_EM_), electro-osmosis (J_EO_), and passive delivery (*J*_p_).^31^


$$J_{DRUG}=J_{EM}+J_{EO}+J_{p}=\left(id\times t_{DRUG}\left|z_{DRUG}\times F\right.\right)+V_{w}\times c_{DRUG}+k_{DRUG}\times c_{DRUG}$$

whereas *id* = Current density applied; *t*_DRUG_ = Transport number of drug particles; *z*_DRUG_ = Charge of the drug particles; F = Faraday’s constant; V_w _= Volume of the solvent flow; *c*_DRUG_ = Concentration of drug; and *k*_DRUG_ = Permeability coefficient of drug.

 Numerous factors of the drug particle, such as pH, atomic size, hydrophilicity, electrode type (preferably Ag/AgCl), application time (maximum 3 min to avoid burns), current strength (physiological limit 0.5 mA/cm^2^), and discontinuous application of current, control the penetration of the drug particle through this method. Among these factors, the molecular size of the drug plays a crucial role in deciding its viability for iontophoresis. It was suggested that the smaller and hydrophilic ions are transferred faster than the larger ions. The permeability coefficients in cationic, anionic, and neutral solutes across the human skin are a function of molecular size. With an increase in the molecular size, there is a decline in the permeability coefficient.^43^ The pH of the solution containing the drug is also a significant factor affecting iontophoretic delivery. Since the pH affects the degree of ionization of the drug, for weak acids, a reduction in pH decreases the ionized portion of the molecules and leads to reduced electromigration.^44^ The current also affects the transfer of particles across the skin. With a rise in the current density, it is anticipated to transfer a more significant quantity of the drug. However, the physiologically tolerable current deemed safe for human use is 0.5 mA/cm^2^ or less. Moreover, in DC, the current is continuous in one direction, while in AC, the current changes periodically.^29^ Lastly, the integrity and thickness of the skin also influences iontophoretic transfer.^45^ Recently this method has effectively distributed human basic fibroblast growth factor, ribonuclease A, cytochrome c, and a variety of high molecular weight particles ( > 12 kDa).^30^

 Park and his co-workers fabricated a device for the synchronized application of sonophoresis and iontophoresis. They reported 240% enhancement in the skin penetration of glutamic acid with the concurrent application of sonophoresis and iontophoresis. This strategy can also enhance the skin permeability of certain cosmeceutical drugs after treatment at a lower intensity and for a shorter period. Moreover, this combinatorial treatment for the physical enhancement of transdermal delivery presents merits such as a reduction in energy density, which reduces skin irritation, thus possessing immense potential for transdermal delivery in the areas of cosmetics and therapeutics.^46^ Fukuta et al achieved the efficient intradermal delivery of antibodies using the non-invasive method of iontophoresis with a weak electric current of 0.4 mA/cm^2^. The antibodies were transdermally delivered from the region extending from the epidermis to the dermis layer. The iontophoretic-mediated transport of the antibodies into the psoriatic inflamed skin tissue was successful, as proven in imiquimod (IMQ)-treated psoriasis model rats. Furthermore, the upregulation of the mRNA levels of interleukin 6, which is involved in the progression of psoriasis, was found to be significantly repressed by the iontophoresis of etanercept. The administration of etanercept through the application of iontophoresis also significantly reduced epidermal hyperplasia in IMQ-treated psoriasis model rats, thereby suggesting the usefulness of this technique for the delivery of biological macromolecular drugs.^47^ Li et al designed an iontophoretic-driven porous microneedle array patch, as shown in Figure 4, for the active transdermal delivery of charged liquid drugs. This technology combined a porous microneedle array and iontophoresis with charged nanovesicles. This drug delivery strategy involves passive diffusion and active iontophoresis of charged nanoparticles encapsulated with a liquid drug. Iontophoresis resulted in the diffusion of the charged nanovesicles stored in the porous microneedle array into the systemic circulation through the interconnecting pores.^48^ An et al developed a strategy for the transdermal drug delivery of electrically mobile drug nanocarriers. The system consists of a portable and disposable reverse electrodialysis battery that can generate electric power for iontophoresis. Furthermore, an electroconductive hydrogel that consists of polypyrrole-incorporated poly (vinyl alcohol) was used to provide a drug reservoir for the iontophoretic process. This work demonstrates that fluconazole- or rosiglitazone-loaded drug nanocarriers could be functionalized with charge-inducing entities and that drug nanocarriers modified with charge could result in facilitated transdermal delivery through repulsive reverse electrodialysis-driven iontophoresis, thereby indicating the non-invasiveness of this technique in transdermal delivery.^49^ The combinatorial delivery of buprenorphine and naltrexone by iontophoresis was performed by Cordery and his coworkers. This method significantly enhanced the flux of both drugs in comparison to the passive mode. A decrease in the current density by increasing the delivery area caused an increase in the electroosmotic flow.^50^ Yang et al created an iontophoresis-microneedle array patch driven with a smartphone for active and controlled insulin administration. The amount of insulin was efficiently controlled by employing varying current intensities. The *in vivo* study revealed a robust hypoglycemic action in a type-1 diabetic rat model with superior control and efficacy than either of the methods alone.^51^

 Despite these merits, a significant drawback of this method is that the delivery and extraction of large entities such as proteins are restricted owing to their inefficient ability to carry the electric charge.^52^ The presence of the analyte in high concentrations in the skin also presents another limitation that necessitates a warm-up period of 2 h or more to achieve a correlation with the subdermal levels.^53^ Although the safety of this method is established, applying high levels of current could cause discomfort, painful burns, skin irritation, and blisters.^54^

###  Electroporation

**Figure 5:**
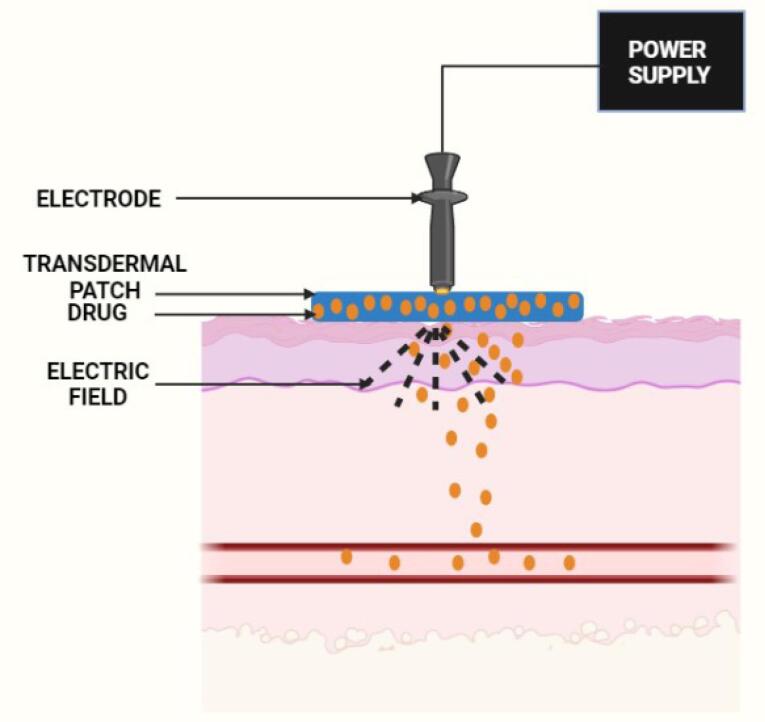
Diagrammatic representation of electrophoresis principle

 This technique uses short and high-voltage electrical pulses of approximately 5–500 V, which, when administered to the skin, results in the development of tiny holes in the SC, enhancing the permeability of the drug particles (Figure 5).^30^

 The principle behind utilizing electroporation for enhancing the percutaneous delivery of drugs is to disturb the barrier characteristics of the SC to promote drug transport.^55^ An external field generates a transmembrane potential in a cell, which is commonly described by the equation:


$$\Delta V_{m}=f\;E_{r}\;\cos\theta$$

whereas V_m_ = Transmembrane potential; ƒ = Geometrical and electrical characteristics of the cell; E = Applied electric field intensity; r = Radius of the cell; and θ = Polar angle with respect to the external field direction.

 Most often, the value of “f” is equal to 1.5.^55^

 The benefits of electroporation are as follows:

- The electroporation criteria can be changed to control the transdermal penetration rate and extent.
- The pores created after applying high pressure are reversible, and the harm is comparatively less.
- The permeability of macromolecules, fat-soluble, water-soluble, or charged particles is better compared to iontophoresis. Electroporation has achieved broad consideration as a viable transdermal method.^31^

Feng and co-workers conducted *in vitro* and clinical experiments to optimize electroporation parameters for the transdermal delivery of sinomenine hydrochloride. The optimized electroporation factors *in vivo* were 3 kHz, exponential waveform, and an intensity of 10, which enhanced the transdermal permeation of sinomenine hydrochloride in miniature pig skin. The process of electroporation-induced transdermal permeation by the electronic pulses caused the skin structure to become looser, thereby enhancing the permeability.^56^ Simon et al recently created a two-in-one electrode material comprising carbon nanotubes and agarose hydrogel. Skin electroporation with unipolar pulses and non-invasive electrodes generated asymmetrical production of aqueous channels in the SC between the side of the electrode. The device showed excellent results of transitory skin permeability and rapid delivery of small molecules into the dermis.^57^

 The electroporation technique also entails several limitations, such as the complicated operating procedure used in the biological field and an inadequate database for clinically significant molecular probes and cells to verify the relevance.^58^ Moreover, the use of pin electrodes makes this method quite invasive.^59^ Another area of concern is the safety of transdermal electrophoresis. Since this technique utilizes high voltage pulses, short-term and long-term safety is a main concern.^60^

###  Microneedles

 Microneedles are microscopic needle-like projections that can penetrate the SC, creating a passageway promoting the easy route of larger molecules.^61^ This route helps in the diffusion of drugs through the dermis layers with a rich abundance of blood vessels. It avoids the stimulation of nerve fibres without damaging the blood capillaries.^62^

**Figure 6:**
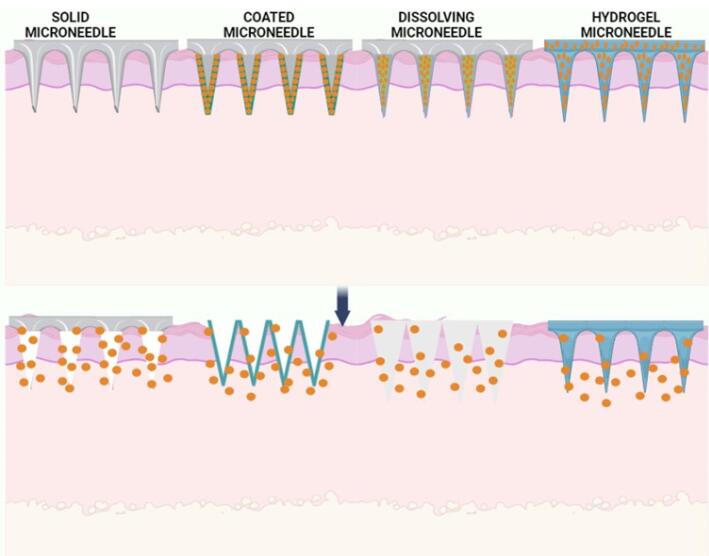
Types of microneedles

 Microneedles are classified based on the drug delivery profile^63,64^ (Figure 6):

- *Solid microneedles:* They follow the ‘poke and patch’ principle. The diffusion of the drug particles/formulation occurs directly into the dermis layer by creating microchannels. Ita et al investigated using cylindrical surface microneedle systemsto deliver medicines for high blood pressure.^63^
- *Hollow microneedles:* These are microstructures that resemble hypodermic needles. The drug is delivered through pressure-driven liquid formulation.
- *Dissolving microneedles:* They function on the ‘poke and flow’ approach. These microneedles are comprised of biodegradable materials, including sugars or polymers, and the medication is loaded in these microneedles.
- *Coating microneedles:* They utilize the ‘coat and poke’ approach.^62^

 Microneedles have found their application in skin cancer and its treatment. They have also been used to study psoriasis, dermatitis, and viral warts. They are also used in ocular treatments such as glaucoma, uveitis, and macular degeneration. Currently available microneedles are used for cosmetic purposes, especially in the treatment of acne.^62^

 Kapoor et al developed an array of coated microneedles to deliver a potent peptide through the transdermal route, which would have a comparable bioperformance in comparison to the subcutaneous method of administration. The solid microstructured transdermal system was precisely coated with two different formulations along with a sub milligram dose of ~250 µg of peptide A on a patch of 1.27 cm^2^ that consisted of 316 microneedles. The stability of the peptide was improved when it was coated onto the microneedle system. Moreover, compared to the subcutaneous liquid formulations, there was negligible degradation at room temperature.^65^ The development of dissolving microneedles involves a reconstitution stage of the drug that could culminate in the degradation of the therapeutics or a limited loading capacity. Thus, Kim et al fabricated powder-carrying microneedles that could self-administer and lack a reconstitution step and instead implanted insulin powder directly into the skin. The developed powder-carrying microneedles showed long-term stability and extended-release kinetics, which presents tremendous potential for treating diabetes without any safety concerns.^66^ In another study, Ahmed and colleagues prepared doxorubicin hydrochloride and celecoxib coloaded liposomes to deliver in combination with derma roller microneedles for the treatment of melanoma. The data obtained from the study indicated that before applying the gel, pre-treatment of the skin with derma rollers greatly enhanced the tumor inhibition rate.^67^

 Yan and colleagues attempted to develop and characterize a dissolvable microneedle patch for the delivery of huperzine A transdermally for the management of Alzheimer’s disease. It was observed from the findings of the *in vitro* transdermal release tests that greater than 80% of the drug cumulatively permeated from the microneedle through the skin within 3 days, thus exhibiting an extended-release profile. Moreover, the *in vivo* pharmacokinetic data indicated that the microneedle group had a greater T_max_, greater t_1/2_, lower C_max,_ and a greater AUC_0-∞_ than the group that received the same oral dose of huperzine A.^68^ Similarly, Kim and his team also investigated a technique for the encapsulation of donepezil hydrochloride in the tips of dissolving microneedles. The micromolding process was used to prepare the microneedles using an HPMC-ethanol/water mixture at 10 °C. A high amount of donepezil hydrochloride was encapsulated within the microneedle tips (up to 78% w/w). Approximately 95% of donepezil hydrochloride was transported into the porcine skin within 5 minutes of insertion due to its distribution in the tips. In comparison to the oral delivery of identical doses of donepezil hydrochloride, the transdermal administration of the drug through the microneedles was found to be more effective, as indicated by the C_max_ and AUC.^69^

 However, the use of microneedles for transdermal delivery also entails specific cons, such as limited drug dose owing to the small size of the microneedle, sophisticated instruments for manufacturing, and appropriate storage containers for distribution from manufacturers to the patients.^70^ Moreover, the likelihood of inflammation in the surrounding tissues and the possibility of the microneedles breaking and being left under the skin, causing an adverse reaction, is also a grave concern.^71^

###  Thermophoresis

 Thermophoresis is a phenomenon in which a particle’s mobility is impacted by a temperature gradient.^72^ The thermophoresis of molecules is greatly influenced by temperature, concentration, ionic strength, mass, and moment of inertia.^73^

 The generation of a temperature gradient can be done in the following ways:

1. *Joule heating is another term for resistive heating:* The resistance of an electrical conductor is applied to express electricity to heat. It is primarily based on Ohm’s law.
2. *Channels:* In microscale devices, a temperature gradient is created by heating or cooling channels. Heat is carried from cold to hot channels in a one-dimensional system. If the thermophysical dimensions of the cold and hot channels are known, their temperature characteristics may be approximated.
3. *Optical heating:* These are used in thermal lens experiments. Optical heating is applied by microscale thermophoresis, in which the infrared laser creates a temperature gradient, and microscale thermophoresis monitors the fluorescently labelled biomolecules and the reaction. Thermophoretic traps are another method that uses heat dissipated from a focused laser beam to monitor nanoobjects without the use of stiff metal nanostructures.

#### Application of thermophoresis^72^

1. Biomolecules accumulate and replicate using thermophoresis and convective flow.
2. To analyse the binding reaction between biomolecules such as proteins and ligands and monitor the binding response.

The significant contribution to these applications is as follows:

- The combination between thermodiffusion and convection leads to the accumulation of drug molecules in the thermophoretic trap.
- When a ligand attaches to the protein, a change in the thermophoresis of the protein takes place, which leads to the detection of the reaction.^73^

###  Skin puncture

 Skin puncture modules are very similar to microneedles. These devices use a needle-like structure to distort the skin barrier by cuts and holes due to the given movement. Skin puncture devices maintain several blades or needles of the desired length, which prevents them from penetrating beyond the surface of the skin.^38^ One such skin perforating device was invented by Kwang K Jang for insulin administration. The device consists of an alternately disposed needle with a spacer combined for rotational movement as a unit in the skin perforating device. It also consists of pair endplates with a diameter more significant than the spacer, which is attached to the device’s opposite end. The primary function of the endplate is to prevent perforation beyond the required depth to avoid pain and flare. Each needle disk has a different number of skin perforation needles, and the alignment of such needles allows the needle to cut more efficiently. After skin perforation, drugs can be delivered as a gel or liquid. This device was introduced to administer medications such as insulin transdermally. Insulin comprises macromolecules with a molecular weight of more than 6000 Da; thus, it cannot penetrate the skin due to its size and hydrophilic property. Before the skin puncture method, insulin was administered through the skin using ointment, patches, and spray. In recent years, the skin perforation method has been considered the best method for transdermally transferring insulin. Another innovative application of skin perforation was introduced by Ciernik et al, in which DNA was introduced inside the body through the skin. This approach allows the installation and expression of plasmid DNA in the skin without the use of a particulate carrier for gene therapy. In this method, skin is punctured with a device consisting of a high-frequency oscillating pack of needles; the DNA transfer is done to skin-associated cells after puncturing.^39^

###  Suction ablation

 This method establishes a suction blister by applying a vacuum or negative pressure. The applied vacuum results in the removal of the epidermal layer, leaving behind the intact basal membrane. This strategy of eliminating skin obstruction is also known as skin erosion. As there is no dermal invasion, discomforts such as bleeding or pain are not seen with this technique. Cellpatch, a product of a Sweden company, is an example of a commercial product that works using this method. This has a cup, epi-dermatome (to shape a blister), and a gadget that can be connected to the skin. In an *in vivo* concentrate by Svedman et al, the conveyance of dextran of different atomic loads (3–70 kDa) and morphine was accomplished through this technique.^74^

 The morphine plasma concentrations were equivalent to those attained through an intravenous mixture. In another investigation, similar creators showed that vasopressin (antidiuretic peptide) accomplished tantamount plasma bioavailability (approx. 100%) to that of direct intravenous imbuement. The expulsion of the epidermal layer by pull caused hyperaemia in the basic dermis. It was identified through laser Doppler flowmetry and confirmed through microscopic techniques. The creators expressed that the noticed hyperaemia could have additionally added to the upgraded permeation noticed. The capability of this technique as a symptomatic instrument has likewise been recently detailed.^75^

 The inconveniences related to the pull technique include the period needed to accomplish a blister (2.5 hours). This can be diminished to a duration of 15 to 70 minutes by warming the skin to 38 °C. Moreover, while there is no danger of fundamental disease contracted and intravenous catheters, the potential for epidermal contaminations cannot be overlooked, even though the impacts may be less genuine.^38^

###  Needleless injection

 Hypodermic needles have been used for more than 150 years to deliver drugs to the body transdermally. However, as science and technology progress, previously used syringes, which are more difficult to sterilize and reuse, are being replaced by newly discovered needleless injections.

 Needle-free injection technology (NFIT) is a novel innovation in transdermal drug delivery systems. This delivery system allows the transfer of the medication through the skin without using hypodermic needles. Instead, forces such as Lorentz, shock waves, pressure by gas, or electrophoresis are used. These devices allow painless and high-efficiency transfer of drugs in contrast to normal syringes.^76^

 NFIT uses an innovative method of transferring drugs through the skin without piercing it. It has shown positive performance in mass immunization and vaccination services due to the painless method of drug transfer.^74^ It is based on the principle that a premeasured dose of a specific medicine formulation, loaded in distinct “cassettes” that can be rigged with the system, is propelled by NFIT using energy powerful enough to accomplish so.^76^

###  Advantages of needle-free injection technology

- There was no skin puncture or any type of damage to the skin.
- Quick drug delivery and improved reproducibility are possible.
- Self-administration is possible.
- Improves vaccine response.
- Medication can also be administered in the form of dry powder.

### Disadvantages of needle-free injection technology:^40^

- The procedure is time-consuming and costly.
- Proper training and upkeep are needed.

In a study by Erlendsson and his co-workers, the delivery patterns of an electronic pneumatic needle-free injection device were studied in porcine skin samples and a basal cell carcinoma tissue sample. From the *in vitro* experiment, it was deduced that the penetration depth increases with an increase in the pressure, whereas the force has a restricted effect on width. Low (30%) and medium (50%) pressure deposits were observed to cover the papillary and reticular dermis, whereas high (65%) and stacking (30 + 50%) pressure deposits covered the subcutaneous dermis. Thus, the drug could be administered using a needle-free injection in a regulated, uniform manner that ensured an immediate and widespread allocation.^77^

###  Photomechanical waves

 Q switch or mode-locked lasers produce photomechanical waves (PWs), which are high amplitude pressure waves. PWs are unique in that they interact directly with the biological target.^34^ Optical breakdowns, ablation, or rapid heating of an absorbing medium are a few techniques through which pressure waves can be produced. The transformation of wave energy and mechanical energy determines the efficiency of PWs.^78^ PW varies from ultrasound waves in that they do not have a negative pressure and therefore do not cause cavitation-induced biological effects.^79^ These unipolar waves promote instant evaporation of water by creating microchannels in the skin, according to the biopsies. In transdermal drug delivery, these waves transiently modify the SC layer by transient pore production without cavitation to increase the permeability in a pain-free manner.^80^ Transient channels are formed as the lacunar mechanism alters, allowing the drug to be transferred into the epidermis and dermis.^36^ Since the PW forces water molecules into these domains, the lacuna system expands, resulting in a continuous path that permits passive drug diffusion across the concentration gradient.^81^ The hydrophilic regions of the lipid structure of the lacuna system are the most vulnerable to such electrical modifications, and this is most likely the driving force behind PW-mediated transdermal drug delivery.^82^

 Parameters for the optimization of PW-mediated transdermal delivery are as follows:

- Wavelength
- Pulse duration
- Optical mechanical properties of the target material applied on top of the skin
- Fluency of laser

PW can be used to transport proteins and peptides in the near future. According to reported animal studies, the maximum size administered to date is 70 kDa.^79^ A study was pursued by Lee and his co-workers, wherein they assessed the influence of the pulse features on the penetration depth of macromolecules transported through the skin. It was observed that in comparison to those produced by direct ablation, PM waves created by constrained ablation of the target have a longer rise time and duration. To raise the depth of delivery from 50 mm to 400 mm for confined ablation, the radiant exposure had to be reduced from 7 J/cm^2^ to 5 J/cm^2^.^80^ PW may be an excellent way to transport macromolecules if further research into their protection and efficacy is performed.

###  Magnetophoresis

 The use of a magnetic field to facilitate drug delivery through the skin is known as transdermal magnetophoresis.^81^ Exposing the skin to a magnetic field may cause structural modifications in the skin, which could lead to an increase in permeability.^82^ A magnetic field is applied around the solute that enters the skin as a part of this enhancing approach.

 Murthy and colleagues attempted to investigate the mechanics of magnetophoretic delivery and to assess if formulating a magnetophoretic patch for lidocaine delivery was feasible. Different magnetic field strengths were used to conduct *in vitro* drug penetration investigations across the porcine epidermis. The “flux enhancement factor” for magnetophoretic drug penetration increased with the strength of the applied magnetic field. “Magnetokinesis” was revealed to be the most common mechanism responsible for magnetically guided drug penetration augmentation. For *in vivo* investigations, a reservoir-type transdermal patch possessing magnetic backing was created. The magnetophoretic patch system’s dermal bioavailability (AUC_0–6_) in rats was considerably greater in comparison to the identically constructed nonmagnetic control patch *in vivo*.^83^ Wright and his research team described a study that employed a crossover design to evaluate a new transdermal product in a quick and reasonably low-cost manner. The goal of osteoarthritis treatment is to alleviate pain and enhance functionality. A novel magnetophoretic technique for transdermal ibuprofen delivery has been developed. To track the pharmacodynamic response to ibuprofen, the researchers employed measurements that were taken over in a short duration. With a five-day washout duration, each subject was randomly administered magnetophoretic-enhanced transdermal ibuprofen or placebo. The volunteers were 24 people diagnosed with painful knee osteoarthritis by a doctor. During the first patch application and for the rest of the research period, there was a substantial decrease in pain associated with movement (*P* < 0.05). The reaction to transdermally administered ibuprofen was a rapid and considerable analgesic response.^84^

 The drawback associated with magnetophoresis is the need for high magnetic intensity and gradient required for adequate control. Only approximately 5 cm of the skin can be focused by stationary magnets of safe intensities, thereby requiring them to be placed close to the diseased region.^85,86^

###  Ultrasound waves

 Ultrasound techniques have been under investigation since the 1950s, when hydrocortisone was used to treat digital polyarthritis. Ultrasonic waves are pressure waves with a frequency of 20 kilohertz, i.e., above the upper limit of the human audible range.^87^

 Acoustic energy is classified into three frequency bands^88^:

- High-frequency sonophoresis: frequencies greater than 3 MHz
- Therapeutic frequency range: 0.7–3 MHz
- Low-frequency sonophoresis: frequencies ranging from 20 to 100 kHz.

The following parameters are taken into consideration while optimizing skin3 permeability using ultrasound waves^89^:

- Frequency
- Wavelength
- Intensity
- amplitude
- Duty cycle
- Application time
- Treatment time
- Distance between skin and tissue
- Composition of coupling medium
- Movement and angle of the transducer

Ultrasound is applied to the skin in three ways^90^:

- *Pretreatment*: Application of ultrasound to the skin before administration of the drug.
- *Simultaneous treatment*: Use of ultrasound through a coupling medium that contains the drug.
- *Posttreatment:* Application of ultrasound after administration of the drug.

Simultaneous treatment and pretreatment are the two most commonly applied methods in a clinical setup. Convection-related mechanisms occur only with simultaneous treatment in the presence of ultrasound, but this is not the case in post- and pretreatment. Drug transport is enhanced because of the structural changes to the skin.^90^

 Several mechanisms of action, such as (1) acoustic cavitation, (2) thermal effects, (3) acoustic radiation and (4) acoustic streaming, have been determined, but the exact method is still not understood.

####  Acoustic cavitation

**Figure 7:**
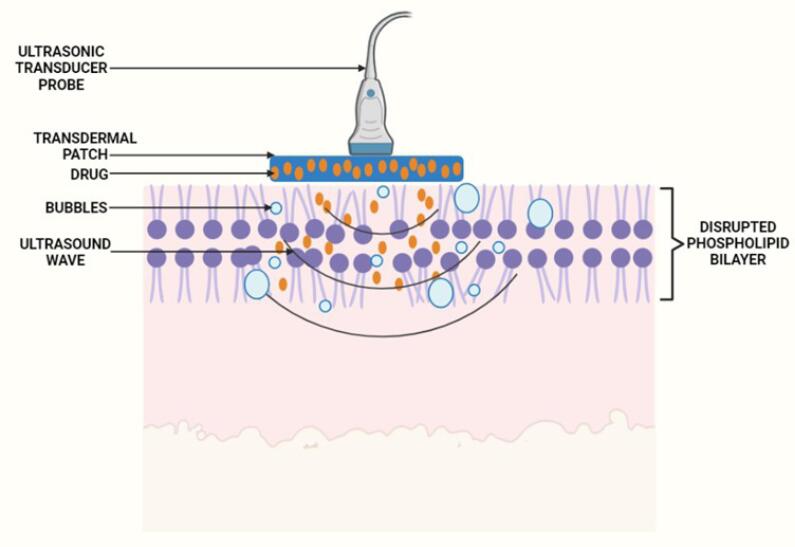
Diagrammatic representation of application of ultrasound waves for transdermal delivery

 The most significant nonthermal effect on biological tissue is thought to be acoustic cavitation. Stable and inertial cavitations are the two forms. In stable cavitation, the bubbles oscillate with an equilibrium radius value without collapsing over time. In inertial cavitation, the bubbles expand as the number of cycles increases and collapse aggressively near the skin.^91^ Ultrasound waves propagate across the coupling medium, causing mechanical vibration of the liquid and acoustic streaming inside the medium, which causes cavitation of gas nucleates.^92^ These bubbles oscillate indefinitely and expand in size over the pressure wave cycles. The scale of the nucleating bubble is proportional to the ultrasound frequency. The bubble size grows as the ultrasound frequency rises, and vice versa. Large bubbles collapse outside the skin when low-frequency sonophoresis are applied, resulting in a jet of fluids (microjets travelling at approximately 500 m/s) that erode dead cells on the SC and permeate the skin. Tiny bubbles alter the permeability of the SC by disrupting the lipid order (Figure 7).

####  Thermal effects

 When subjected to an ultrasound wave, particles in biological tissue oscillate because of the mechanical energy. Some of this energy is transformed to heat, thereby elevating the skin’s temperature. As the temperature rises, the kinetic energy and diffusivity of the drug molecules increases, as does the dilatation of sweat glands and hair follicles (entry points of the skin). The blood flow improves, allowing the drug compounds to circulate more effectively in the body. Thermal effects are proportional to the service cycle and frequency strength.^90^

####  Acoustic radiation

 When an object is positioned in the path of an ultrasound wave, energy is transferred, causing the object to travel in the course of the wave’s transmission in regions with the highest ultrasound pressure amplitude. Acoustic radiation has a widespread range of uses in tissue engineering due to its capacity to transfer cells, microbubbles, and other particles into tissues.^93^

 Acoustic radiation has two forces. The primary force of acoustic radiation is responsible for pushing the particle away from its acoustic source. In contrast, secondary forces attract particles such as ultrasound contrast agents (agents used to increase local drug diffusivity across biological membranes) such as Imagent, SonoVue, Optison etc.^94^

####  Acoustic streaming

 Ultrasound is a nonlinear wave that induces oscillatory regulatory motion and a second-order effect of wave propagation that creates a time-dependent flow velocity that causes the fluid to travel in a specific direction towards the skin surface. Reflection and distortion of wave propagation are the resultsof acoustic streaming.^95^ As acoustic cavitation occurs, the cavitation area is filled with streaming currents and tremendous shear stress due to violent bubble collapse.

 Aside from transdermal drug delivery, ultrasound waves are commonly used in surgical tissue cutting and hemostasis, sonophoresis, sonoporation, targeted gene therapy, bone fracture healing promotion, and thrombolysis. To increase the permeation of diclofenac through the skin, Huang and his co-workers formulated a transdermal system consisting of a polyamidoamine dendrimer combined with sonophoresis. The ultrasound application time was found to substantially influence the *in vitro* drug release. The cumulative quantity of drug permeated through the skin after 24 h was 257.3 µg/cm^2^ after application of the diclofenac dendrimeric gel without sonophoretic treatment. On the other hand, when the gel was applied to skin that was subjected to sonophoresis, the amount of drug that permeated through the skin increased to 935.21 µg/cm^2^.^96^ Yin and his co-workers determined enhancement in the percutaneous permeability of sinomenine hydrochloride by using sonophoresis at 20 and 800 kHz. The results of the research showed that a single-frequency as well as dual-frequency significantly enhanced skin permeability in comparison to the control. The cumulative amount of sinomenine delivered through the skin on the application of dual frequency after 12 hours was 4.3 times and 8.3 times greater than the amount delivered on the application of a single frequency. The greater cumulative amount of drug permeated on the application of dual frequency could be attributed to the increase in transient cavitation instead of simple energy superposition. Thus, the method of dual-frequency sonophoresis for transdermal transport was found to be a potential method to enhance the accumulative amount of the drug and negate the thermal side effects.^97^ Pamornpathomkul et al evaluated the combinatorial use of a microneedle patch and low-frequency sonophoresis to deliver water-soluble macromolecules into the deep skin layers. The current study utilized excised porcine skin to optimize the parameters that affect the delivery of fluorescein isothiocyanate dextran (FD-4). The *in vitro* permeation experiment demonstrated that the extent of permeation was greater for the combination of microneedles and sonophoresis than for each method alone.^98^ Manikkath et al aimed to evaluate the individual and collective impacts of peptide dendrimers and low-frequency ultrasound on the permeability of transdermally administered ketoprofen. The results of the study indicated that combining peptide dendrimer treatment with ultrasound application exhibited enhancement ratios of up to 1369.15. The *in vivo* analyses also confirmed that dendrimer and ultrasound-assisted permeation of the drug resulted in a greater plasma concentration of the drug in comparison to passive diffusion alone. Therefore, this experiment revealed that peptide dendrimers with a terminal arginine group in combination with sonophoresis could substantially enhance the permeation of ketoprofen transdermally.^99^

 One of the disadvantages associated with ultrasound delivery is the requirement of a pre-treatment protocol since the application of the ultrasound device constantly to the skin during simultaneous treatment could result in the degradation of the drug molecule in the coupling medium.^100^ Additionally, the kinetics and amount of the drug that can be transported through the transdermal route, the size and cost of instruments, and long-term safety studies pose major challenges for this technique in the future of transdermal delivery.^101,102^

###  Radio-frequency microchannels

 Cell ablation with a radio-frequency (RF) alternating electrical current is a popular method that could be used to counteract the limitation in the types of active molecules that can be administered by passive transdermal drug delivery. Radio-frequency microchannels (RF-MCs) have been touted as a viable transdermal delivery technology for injectable hydrophilic molecules.^103^ The life span of the microchannels is a significant consideration when evaluating this technology. RF ablation is a renowned medical technique for eliminating live cells.^104^ It is extensively employed in minimally invasive procedures to cut through tissues and remove tumors in the liver and kidney. In this method, a conducting wire is placed on an area of the body, and an alternating electric current is transmitted at a frequency greater than 100 kHz. The ions present in the cells close to the electrodes vibrate as they continue to trail the shift in the path of the electrical current. The heat generated by these vibrations causes the water to evaporate, resulting in cell ablation. RF microchannels are generated when a densely packed series of small electrodes are placed with highly accurate measurements against the skin. Each microelectrode conducts an alternating electrical current that ablates the cells beneath it and generates microscopic channels in the SC and outer dermis. Outer layers of the skin, which are devoid of blood vessels or nerve endings, are penetrated by these RF microchannels. The time window in which the MCs remain open impacts the delivery rate and effective delivery length.^103^

 Ahn and his team developed a microporation instrument based on radiofrequency to improve transport efficiency by eliminating the epidermal layer. The microporation device created micropores on pig skin and human cadaver skin with the dermis and epidermis. By using a Franz diffusion cell, the increased permeability through micropores was verified. Both pig and human skin FITC-reduced dextran’s molecular weight allowed for greater penetration. Additionally, pig skin has a higher incidence of permeation than human skin.^104^ The experiment performed by Park and his coworkers set out to determine how fractional RF and sonophoresis affected the skin’s ability to absorb 5-aminolevulinic acid (ALA). Male domestic pig skin was subjected to fractional RF and/or sonophoresis before receiving topical ALA. After fractional RF, the fluorescence intensity rose in the epidermis and dermis, increasing even more when sonophoresis was added.^105^

 A few patents available on the physical permeation techniques have been included in Table 2.

**Table 2:** Few available patents on different techniques of transdermal drug delivery systems

**Therapeutic Moiety**	**Technique utilized**	**Invention**	**References**
Iron	Iontophoresis	An iontophoretic patch for transdermal delivery of a therapeutic amount of iron, wherein the patch comprises an electrode and reservoir that consists of a composition of ionic iron.	^106^
Insulin, human growth hormone	Iontophoresis	An effective method for transdermal delivery of pharmaceutical agents through the formation of microchannels and further delivery of the drugs by iontophoresis through these microchannels.	^107^
Steroids corticosteroids, analgesics or COX-2 inhibitors	Microneedles	The device includes a sheath bandage placed on the wrist to deliver high therapeutic local concentrations of an anti-inflammatory drug using microneedles that delivers the drug to the wrist joint tissues and the synovial cavity.	^108^
Pharmaceuticals, cosmeceuticals and/or nutraceuticals	Microneedles in combination with iontophoresis or electroporation	An active transdermal patch driven by a programmable processor allows for the application of a combination of energy sources that propel the delivery of drugs with synergistic permeation techniques. Thus, it enhances the permeability of small and large molecules together with providing a controlled transdermal drug delivery system.	^109^
Viral vectors, DNA, chemicals, drugs and/or proteins	Electroporation	A pulsed electrical energy source creates a cold plasma, wherein the cold plasma bears a pulsed electrical energy field that results in a controlled state for electroporation in the target cells. Subsequently, the enhancement of permeability of the cell membrane allows the entry of the drug or chemical into the target cells.	^110^
Neurotoxin	Ultrasonic energy	An active portion of the botulinum toxin, like the light chain fragment of botulinum toxin serotype A can be delivered for facilitating the disruption of the neurogenic activity of the promoters of syndromes that have an underlying neurogenic constituent. When the light chain fragment of botulinum toxin is applied in the presence of an electric field or ultrasonic energy, it results increase in the permeability of target cell wall that induces reversible pore formation of the cell membrane and effective delivery of the light chain fragment to the catalytic surroundings of the cell cytosol.	^111^
Serotonin receptor antagonists e.g., ondansetron, palonosetron	Microneedle	This invention describes a microinjection that comprises a microneedle array and a serotonin receptor antagonist formulation which includes ondansetron or palonosetron containing formulations for the treatment of nausea or vomiting.	^112^
Guanethidine	Iontophoresis	An iontophoretic delivery system for the denervation of the renal sympathetic nerve. The device included a catheter fixed with a drug coated balloon. The operation of the drug-delivery catheter creates an electric potential gradient within the adjacent tissue, which subsequently promotes iontophoretic delivery of the drug.	^113^
Preferred therapeutic agents are calcitonin, desmopressin, goserelin, leuprolide, vasopressin, buserelin, triptorelin, interferon alpha, interferon beta, interferon gamma, glucagon, LHRH, LHRH growth, FSH, EPO, GM-CSF, G-CSF, IL-10, releasing factor and analogues.	Microprotrusions	Invention is around a device and method for the delivery of potent therapeutic agent through SC of mammal by coating a plurality of SC-piercing microprotrusions.	^114^
Rivastigmine, fentanyl and rotigotine.	Transdermal Patch	Invention describes the transdermal delivery of tertiary amine drugs for extended period of time, i.e., for 3 days, 7 days or more.	^115^
Analgesics, anti-arthritic, anti-asthmatic, anti-convulsants, anti-depressants, anti-dotes, anti-viral, anti-inflammatory, etc.	Transdermal patch	Invention pronounces the development of transdermal patch plus microneedle-based arrays including a combination of energy sources (viz., heat, sound and electricity) such as iontophoresis or electroporation to enhance the skin permeation of small as well as large molecules for local and systemic delivery of drugs.	^116^

## Conclusion

 This analysis aimed to give readers an in-depth look at the various physical methods of permeation enhancement employed in transdermal drug delivery methods. These methods are also distinct from the chemically dependent approach. According to the report, several methods are available but seldom used. A few methods are widely adopted in markets, and some are seldom used due to their drawbacks. Transdermal drug administration technology is increasingly filling up the pharmaceutical industry by evolving medicinal technologies. It is fruitful in offering significant commercial demand because it is a simple route of administration capable of supplying even substantial doses over extended timeframes. New approaches for developing and commercializing transdermal drug delivery technologies, such as the use of electromechanical, acoustics, sound, friction, microneedle amplification, and heating strategies for the preparation and design of new products, such as 3D printing of transdermal medications, enhanced performance patches, and fast and non-invasive infusion applications, are changing the situation. Physical methods are widely used to treat discomfort. Various trials have shown the versatility of dosage administration, long-term pain management, and minimal adverse effects. Clinical trials in this area of research are currently in high demand. Manufacturing prices for these surgical instruments and the high expense of these procedures must be resolved. In the future, it could be feasible to merge electrically and chemically regulated processes with physical methods to remove the drawbacks of each procedure separately while also making it more effective and swifter in operation. To accelerate the use of enhancement techniques, especially physical approaches, considering their numerous benefits, studies on human subjects are required to comprehend the mechanism of drug delivery and a deep understanding of these cutting-edge techniques. Many researchers are leading the charge to move experimental studies into clinical trials, as this would pave the way for the future of transdermal delivery, given the introduction of handheld devices further to improve the treatment of chronic illnesses with such techniques.

## Competing Interests

 The authors declare that they have no competing interests.

## Ethical Approval

 Not applicable.
